# *De Novo* Presentation of Systemic Lupus as Bullous Erythematosus: A Case Report

**DOI:** 10.2174/0115733971341840241206080539

**Published:** 2024-12-09

**Authors:** Saima Majid Mattoo, Sarah Mohammed Iftikhar, Rajesh Gupta Gopal, Imadeldin Ahmed Hamad, Shubhada Mandar Bichu

**Affiliations:** 1 Department of Internal Medicine, Kuwait Hospital Sharjah, Sharjah, UAE;; 2 Department of Dermatology, Qassimi Hospital Sharjah, Sharjah, UAE

**Keywords:** Bullous systemic lupus erythematosus, systemic lupus erythematosus, bullous lesions, case report, conclusive diagnosis, *de novo*

## Abstract

**Background:**

Systemic Lupus Erythematosus (SLE) (C1) is a disease with multi-organ involvement that can have a variety of cutaneous manifestations in 76% of cases during the disease. Less than 1% of these patients are diagnosed with confirmed bullous systemic lupus erythematosus (C1). Given the wide differential diagnosis of a bullous lesion, it is imperative to reach a conclusive diagnosis as it can have a direct impact on the course of management of the disease. Here, we present a case of SLE with a *de novo* presentation of bullous lesions. Throughout the length of the report, we will go through the protracted clinical course of the patient, followed by a clinically relevant discussion of the condition.

**Case Presentation:**

The case describes the presentation of a young African female of low socio- economic status with first-ever eruption of bullous lesions on her trunk and groin. The lesions progressed to involve the face. A biopsy was taken, and the patient was started on dapsone and hydroxychloroquine. She initially responded well but soon developed Steven Johnson syndrome in reaction to dapsone. In the meantime, a biopsy and hematological work-up confirmed a diagnosis of Bullous SLE. The patient was started on methotrexate, to which she initially responded well but developed methotrexate-induced cytopenia. This was followed by initiation of mycophenolate, to which the patient responded very well and was subsequently discharged on the same. At the time of discharge, all lesions healed, and the hematological workup remarkably improved.

**Conclusion:**

All patients with bullous lesions should be evaluated for bullous SLE. A definitive diagnosis will chart the course of management. Multiple drug options are available, and there is no single hierarchy of medicines that will suit all. Sometimes, multiple modalities need to be tried before the patient achieves clinical remission and then can be continued on the same.

## 1 INTRODUCTION

Systemic Lupus Erythematosus (SLE) is a disease with multi-organ involvement that can have a variety of cutaneous manifestations in 76% of cases during the course of the disease. Of these, less than 1% of patients can be identified as having definite bullous Systemic Lupus Erythematosus (C1) [1]. Bullous SLE is a rare auto antibody-mediated manifestation of SLE, which causes the development of subepidermal bullous lesions, mostly in young females of African descent.

It is also important to monitor patients presenting with SLE and bullous skin lesions as they can develop full systemic disease with time. We report a case of SLE with a *de novo* presentation with bullous lesions all over the body, including the face, chest, and limbs. The oral mucus membrane, palms of hands, and soles of feet were spared. Lesions were most fulminant on the trunk and perineal area.

## 2 CASE REPORT

Our case report is about a 33-year-old female of African descent. She is an expatriate, living on a resident visa for the last 1.5 years. Her family lives in her hometown in Ethiopia. The patient works as a salesgirl in a supermarket. There is no positive family history of any chronic medical illness (C3).

**Fig. (1):**
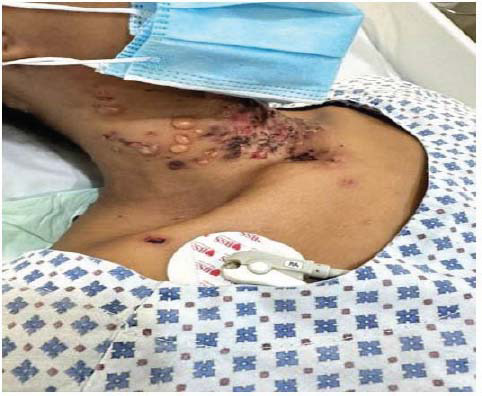
Lesions in various stages-Bullous lesions with underlying pigmentation on the neck.

**Fig. (2):**
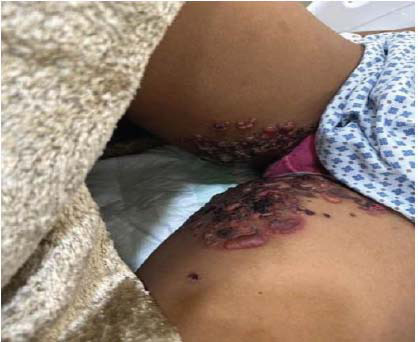
Bullous Lesions with scattered pigmentation on the upper thigh.

**Fig. (3):**
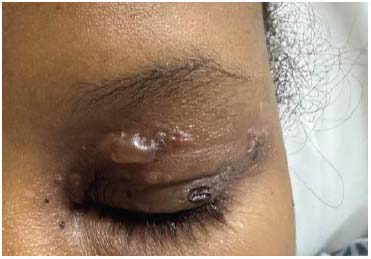
Thin-walled bullous Lesion on eyelid.

The patient presented to the emergency department of our facility with a 1-week history of fever and bullous lesions all over the body. She denied any past medical illness. Clinical examination revealed thin-walled, tense bullous lesions all over the body, including the face, chest, and limbs. The oral mucus membrane, palms of hands, and soles of feet were spared. Lesions were most fulminant on the trunk and perineal area (Figs. **1**-**3**).

On presentation, the patient was febrile with a temperature of 38 degrees. She appeared pale. The chest examination was normal. A cardiovascular examination showed muffled heart sounds. There was no peripheral edema. Initial laboratory investigations showed low hemoglobin, low white cell count, low albumin, and a CRP of 21 (Table **1**).

Based on the clinical picture and with an initial impression of bullous dermatosis, the patient was started on oral and topical steroids as well as oral and topical antibiotics. At this point, our investigations are targeted toward ruling out autoimmune diseases like bullous pemphigoid and SLE.

Autoimmune workup was sent, the results of which were as follows (Table **1**). Initial complement C3 and C4 levels were low as well.

**Fig. (4):**
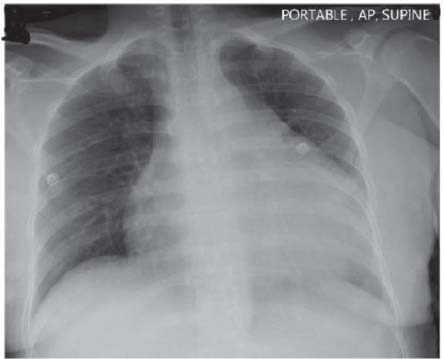
Initial Chest x-ray showing increased cardiothoracic ratio.

Chest Radiographic Examination showed evidence of increased cardio-thoracic ratio (Fig. **4**). Echocardiography showed Moderate pericardial effusion without evidence of chamber collapse or hypertensive heart changes.

**Fig. (5):**
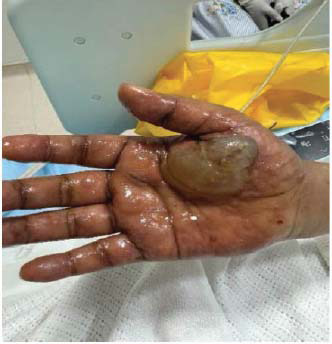
Dapsone-induced Steven Johnson syndrome (Hand) causing large blister-like lesion.

**Fig. (6):**
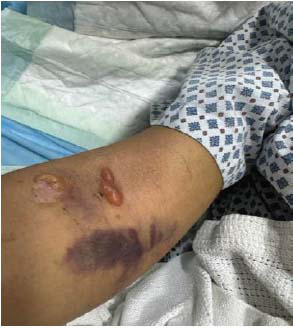
Dapsone-induced Steven Johnson syndrome (Forearm) causing blistering lesion.

**Fig. (7):**
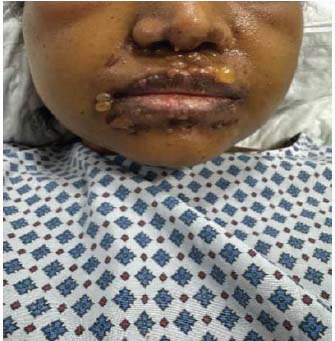
Dapsone-induced Steven Johnson syndrome (Face) causing blistering lesions.

**Fig. (8):**
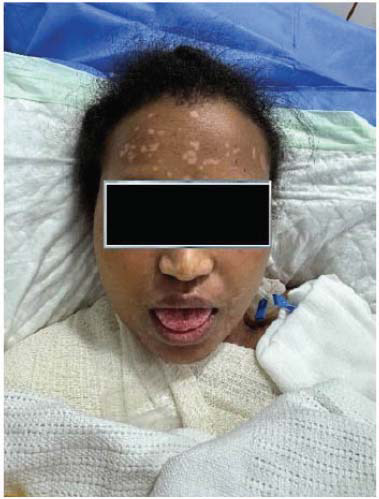
Healing process (Face)- Previous lesions have healed.

**Fig. (9):**
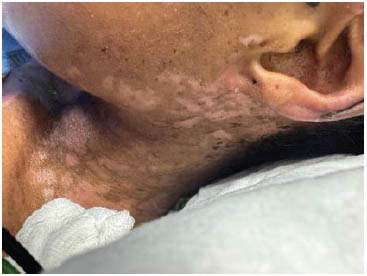
Healing process (Neck)- Previous bullous lesions have healed, leaving behind hypo pigmented macular patches.

**Fig. (10):**
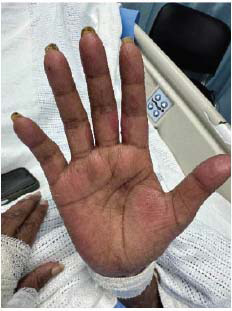
Healing process (Hand)- All lesions have healed, and the skin surface looks healthy again.

**Fig. (11):**
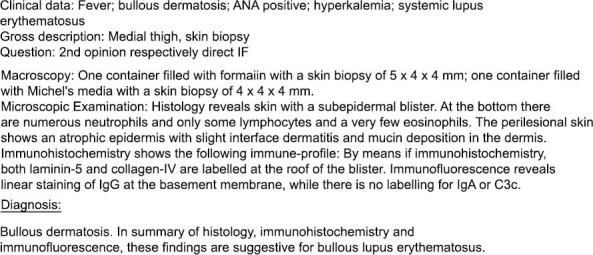
Histopathology report.

Based on the clinical picture and investigation findings of positive ANA, pancytopenia, DsDNA, low C3 and C4, and pericardial effusion, the diagnosis of bullous SLE was formulated according to American College of Rheumatology criteria for classification SLE (12) (C4). A biopsy of the lesion on the upper thigh was performed, and histopathological diagnosis was confirmed to be bullous SLE (Figs. **5**-**11**).

The patient was started on hydroxychloroquine 400 mg daily and prednisolone 60 mg (C5) initially. Dapsone was added after confirmatory diagnosis and after ruling out G6PD deficiency. Patients started showing rapid clinical improvement after initiation of dapsone. However, unfortunately, a few days after starting dapsone, the patient started developing painful lesions on her face, hands, and oral mucosa. The involvement of oral mucosa was not observed in the initial presentation. The clinical picture was consistent with Steven Johnson syndrome (Figs. **5**-**7**). Dapsone was immediately withdrawn. The patient was started on immunoglobulin, after which clinical improvement in the Steven Johnson symptoms was observed. Oral lesions did not recur at any time after that. No biopsy was done on these lesions. Methotrexate was initially given in the dose of 10 mg/kg weekly and slowly increased to 15 mg/kg. After 1 month of use of methotrexate, patients started developing bicytopenia involving a significant reduction in white cell count (from 4.09 to 1.15) and red cell count (3.24 to 2.73). This led to the cessation of methotrexate as a treatment modality. Complement levels were again checked and found to be persistently low, indicating the disease activity. Mycophenolate started at a dose of 1 gm twice a day and increased subsequently to 1.5 gm twice a day. The patient has been improving since then. Her skin lesions have completely healed with residual hypopigmentation (Figs. **8**-**10**). Although complement levels are still low, her white cell counts and hemoglobin levels improved (Table **2**). She has been taking prednisolone 5 mg daily (C5) and mycophenolate 1.5 mg twice a day and has achieved clinical remission. The patient stayed in our facility for 2 weeks after this period. She traveled back to her home country after that and hence could not be followed in the outpatient department. (C6).

## 3 DISCUSSION

We presented a case of a young female with *de novo* presentation of bullous SLE, which needed more than one modality of treatment before successful remission with mycophenolate (C8). Bullous SLE is a rare auto antibody-mediated manifestation of SLE, which causes the development of subepidermal bullous lesions, mostly in young females of African descent [2]. It is mostly diagnosed through histological investigation and is associated with high levels of circulating antibodies to Type VII collagen. Lesions tend to appear in sun-exposed areas. Mucosal involvement is common, though not absolute, as seen in our presented case. Depending on immunopathological findings, Bullous SLE can be classified into three types based on the target of autoantibodies [3-5]. Type 1 bullous SLE is the commonly seen subtype with autoantibodies targeting type VII collagen, non- collagenous 1 and 2 (NC-1 and NC-2) domains. NC-1 plays a vital role in maintaining the integrity of the dermal-epidermal junction. As per Camissa and Sharma's criteria, diagnosis of Bullous SLE requires the fulfillment of at least 4 of the following 5 criteria (4, 5 and 2).

- Confirmed diagnosis of SLE as per American College of Rheumatology criteria.
- A new onset vesiculobullous eruption.
- A subepidermal blister with predominantly neutrophilic infiltrate is seen in histopathology.
- Deposits of Immunoglobulin G (IgG) with or without Immunoglobulins A or M (IgA and IgM) at the basement membrane, as seen on direct immunofluorescence.
- The presence of antibodies to Type VII collagen on direct or indirect immunofluorescence.

A further criterion was added by Gammon and Briggaman with the advent of electron microscopy. This includes co-distribution of immunoglobulin deposits with anchoring fibrils/ Type VII collagen. Gammon and Briggaman further narrowed down the classification to two classes only, with Type I satisfying all 6 criteria and Type II satisfying the first 4 criteria. Given the bullous nature of lesions, the differential diagnosis of bullous SLE is wide. In the below table, we have attempted to tabulate the comparative parameters amongst the most common differentials (Table **3**) [6].

For our patient, the diagnosis was clear by histopathology. The patient initially showed positivity for all SLE markers along with hematologic and renal involvement. Repeat workup after the treatment initiation showed resolution of most of the parameters. Only the complement level remained low, indicating the active nature of the disease.

Even though hydroxychloroquine treatment is useful in all patients of lupus and should be used unless contraindicated, dapsone is the single most effective drug used in the treatment of bullous SLE, leading to a resolution in 90% of cases. Steroids are used in patients where dapsone is contraindicated. Meanwhile, methotrexate and mycophenolate are used as steroid-sparing agents [7-10]. Rituximab and short-course immunoglobulins are emerging treatment options. Our patient was initially treated with dapsone with a good response but needed to switch to steroids and mycophenolate as she developed Steven-Johnson syndrome. An excellent response was obserevd in our case with mycophenolate, and she remained stable on a maintenance dose.

Although statistical analysis was not done in this case (being a single case report) , but improvement in certain markers are clearly visible as depicted in Fig (**12**).

Irrespective of underlying disease activity, bullous SLE is usually transient. Recurrence has rarely been reported. An extensive review of the literature provided only two such instances. Lesions heal generally without milia but may leave residual hyper or hypopigmentation. Both sequelae were observed in our patient.

Whenever a patient with a variegate diagnosis of systemic lupus erythematosus presents, it is important to address every aspect of the disease. Our patient presented with skin lesions predominantly. The treatment would be incomplete if we did not address complaints related to other systems. Hence, a holistic approach is needed for such patients. In addition to exhaustive treatment by the medical team, the patient was also referred to a wound care team, cardiologist, pulmonologist, psychiatrist, and dietitian team to address the co-morbidities.

### 3.1 Medication Details

The following treatment options, almost all of which were used for our patient in a step-by-step manner, can be used to treat bullous SLE.

#### 3.1.1 Hydroxychloroquine

Hydroxychloroquine treatment is useful in all patients of lupus and should be used unless contraindicated.

##### 3.1.1.1 Mechanism of Action

Exerts anti-inflammatory and immunomodulatory actions by mechanisms not clear.

##### 3.1.1.2 Dose

400 to 600 mg per day in 1 to 2 divided doses.

##### 3.1.1.3 Side Effects

Allergic reactions, increased incidence of retinopathy with higher doses.

#### 3.1.2 Dapsone

This is the single most effective drug used in the treatment of bullous SLE, leading to resolution in 90% of patients. Most patients stop forming new blisters almost immediately, and healing of preexisting blisters takes place within a few days.

##### 3.1.2.1 Mechanism of Action

Inhibition of polymorphonucleocytes and complement activation, which leads to blistering in SLE.

##### 3.1.2.2 Dose

25-50 mg per day.

##### 3.1.2.3 Side Effects

Hemolytic anemia, agranulocytosis, drug-induced bullous disorder (like Steven Johnson syndrome, as in our case).

##### 3.1.2.4 Caution

Pregnancy category C, G6PD deficiency.

#### 3.1.3 Corticosteroids

These are most beneficial in patients for whom dapsone is contraindicated, who cannot tolerate dapsone due to side effects, or who have other systemic manifestations of SLE. For our case, we used dapsone along with steroids from the outset due to evidence of systemic disease. Enteral and parenteral corticosteroids can be used.

##### 3.1.3.1 Mechanism of Action

Suppression of pro-inflammatory cytokines, leading to improvement in bullous lesions in SLE.

##### 3.1.3.2 Dose

0.5 to 1 mg/kg/day of prednisolone.

##### 3.1.3.3 Side Effects

Long-term side effects of corticosteroids like weight gain, hypertension, osteoporosis, muscle cramps, skin thinning, acne.

#### 3.1.4 Methotrexate

Used as a steroid-sparing agent or alternative to dapsone when the patient cannot tolerate it because of side effects or contraindications. Methotrexate helps reduce steroid use by up to 44% in patients with SLE needing chronic steroids.

##### 3.1.4.1 Mechanism of Action

Methotrexate improves symptoms of inflammation by increasing the concentration of adenosine (anti-inflammatory) at sites of inflammation.

##### 3.1.4.2 Dose

7.5 mg/week oral, titrated to achieve maximal response. It can also be given as 2.5 mg every 12 hrs. for 3 sequential doses per week.

##### 3.1.4.3 Side Effects

Leukopenia, thrombocytopenia, agranulocytosis, anemia, azotemia, gingivitis, hyperuricemia, nausea, vomiting, diarrhea.

In our case, methotrexate had to be stopped due to leukopenia and anemia.

#### 3.1.5 Mycophenolate Mofetil

Used as an immunosuppressant. Since the pathology of bullous SLE involves autoantibody response leading to disruption of dermo-epidermal junction, mycophenolate can be used to suppress this response and achieve remission. An excellent response was observed in our case with this medication, and she remained stable on a maintenance dose.

##### 3.1.5.1 Mechanism of Action

Mycophenolate inhibits T and B cell production and autoantibody production as well.

##### 3.1.5.2 Dose

1 gm Twice a day, can be titrated upward to a maximum dose of 3 gm/day in 2 to 3 divided doses.

##### 3.1.5.3 Side Effects

Hyperglycemia, electrolyte disturbances, increased urea, leukopenia, anemia, fever, infection, diarrhea, nausea.

##### 3.1.5.4 Caution

Increased susceptibility to infections, red cell aplasia when used in combination with other immunosuppressants, pregnancy category D (Double contraception recommended).

#### 3.1.6 Rituximab

Emerging as a treatment of choice for patients with bullous SLE. Rituximab is a biological agent that targets many of the key components involved in the production of symptoms in bullous SLE [16].

##### 3.1.6.1 Mechanism of Action

Rituximab is an anti-CD 20 monoclonal antibody that decreases anti-Type VII collagen antibodies by depleting mature B cells.

##### 3.1.6.2 Dose

1 gm every 2 weeks.

##### 3.1.6.3 Side Effects

Hypersensitivity reactions, agranulocytosis, myalgia, asthenia, back pain, cough, infection, night sweats.

##### 3.1.6.4 Caution

Fatal infusion reactions, severe mucocutaneous reactions, reactivation of hepatitis B virus.

#### 3.1.7 Other Modalities of Treatments

Extensive lesions may be treated by a short course of IV immunoglobulins, especially in dapsone allergic patients. Thalidomide and lenalidomide have been seen to be effective in patients with SLE with cutaneous lesions. However, their efficacy in bullous SLE still remains unknown [11, 12]. Cyclophosphamide, especially when used as pulse therapy, has shown success in treatment of lupus disease in general, especially lupus nephritis. It has also shown benefit in resolution of pulmonary, cardiac, hematological and neuropsychiatric disease manifestation. Use in bullous SLE is not well documented but is subject of consideration and research [13]. IV immunoglobulin has been used as sole therapy as well as in combination with mycophenolate has also shown promising results in treatment of patients with severe bullous SLE [14-16].

#### 3.1.8 Biologic Treatment

Anifrolumab, which is a human monoclonal antibody to type 1 interferon receptor, has been recently approved for treatment of lupus in steroid dependent patients. In phase 3 trials, a significant proportion of patients were seen to achieve remission. Specific response in bullous SLE is not yet known as this drug is still not in wide usage [14].

## 4 PROGNOSIS

Irrespective of underlying disease activity, bullous SLE is usually transient. Recurrence has rarely been reported. An extensive review of the literature provided only two such instances. Lesions heal generally without milia but may leave residual hyper or hypopigmentation. Both sequelae were observed in our patient.

## CONCLUSION

Bullous SLE needs to be included in differential diagnosis of any kind of skin rash. To get the maximal response to treatment, the most important step is to first diagnose the condition definitively. The advent of CD-20 inhibitors and plasmapheresis for treatment of SLE have been game changing for refractory cases. This case has been presented for its rarity of this disease and showed good clinical improvement with early diagnosis and treatment. Although findings cannot be generalized due to rarity of this condition, we can nevertheless definitively conclude that more than one modality of treatment may need to be implemented to get successful remission.

## AUTHORS’ CONTRIBUTIONS

The authors confirm their contribution to the paper as follows: study conception and design: SMM; data collection: SMB; analysis and interpretation of results: IAH, RGG; draft manuscript: SMI. All authors reviewed the results and approved the final version of the manuscript.

## Figures and Tables

**Fig. (12):**
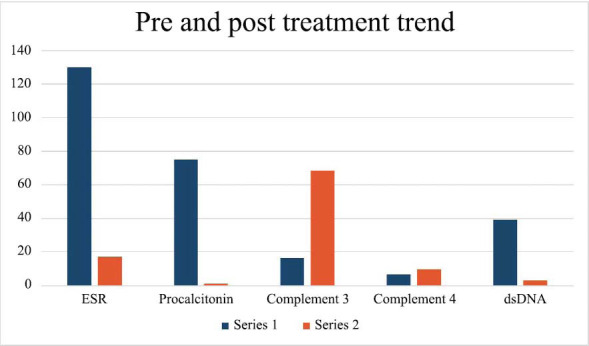
Pre and post treatment trend of markers.

**Table 1:** Initial Laboratory work up (C10).

**Test Name**	**Result**
WBC	3.55×10E3/ul(low)
Hemoglobin	8.30g/dl(low)
Platelet	118×10(3)/mcL(low)
INR	1.00 ratio
Sodium	134mmol/L
Potassium	4.75mmol/L
Creatinine	73umol/L
Urea	6.87mmol/L
Albumin	23.4g/L(low)
Bilirubin total	7.9umol/L
ALT	14 IU/L
AST	29 U/L
Alk phos	43.04 IU/L
Total CK	19 IU/L
CRP	21.9mg/L
SS-A	Positive
RNP/Sm	Positive
Sm	Positive
SS-A	Positive
RNP/Sm	Positive
Sm	Positive
DNA Ab (DS)	Positive
ANA	Positive

**Table 2:** Laboratory results post mycophenolate.

**Detail**	**Value w/Units**	**Flags**	**Normal Range**
ESR	39.00 mm/hr.	HI	0.00-20.00
C3 Complement	51.2 mg/dL	LOW	90.0-180.0
C4 Complement	9.6 mg/dL	LOW	10.0-40.0
CRP	97.4 mg/L	HI	0.0-3.0
DNA Ab (DS)	Negative	NA	-
WBC	5.43 x10E3/Ul	-	4.00-10.00
Hgb	8.70 g/dL	LOW	12.00-15.00
Platelet	272.0 x10(3)/mcL	-	150.0-450.0
PCT	0.06 ng/mL	-	0.00-0.10

**Table 3:** Differential diagnosis.

**Parameter**	**Bullous SLE**	**Bullous Pemphigoid**	**Pemphigus Vulgaris**	**Toxic Epidermal Necrolysis**	**Linear IgA Dermatosis**
Nature of bulla	Tense bulla/urticarial plaques in sun-exposed areas heal with residual hypopigmentation	Tense bullae/urticarial rash, heal with milia, no scarring	Flaccid blister involving mucosa breaks easily, leaving behind a crust	Erythematous macules with dark center, flaccid bullae, Nikolsky sign positive	Tense blisters in string of pearls appearance
Autoimmune profile	Positive markers for SLE, Auto antibody against Type VII collagen	Positive BP 180 (Type XVII collagen) or BP 230	Circulating IgG antibodies against desmoglein 1 and 3	No specific blood tests or history, is important; Cytokines may be seen, along with drug-specific CD8^+^ cytotoxic lymphocytes, in early blister fluid	None in particular
Histopathology	Subepidermal blister with neutrophilic infiltrate and deposition of IgG (with or without IgM and IgA) at the basement membrane	Subepidermal bulla with neutrophils and deposition of IgG + C3 (with or without IgM and IgA) at the basement membrane	Linear/granular deposition of IgG and C3 in epidermal intercellular spaces	Keratinocyte necrosis on histopathology, Direct immunofluorescence is negative	Subepidermal bulla with neutrophils in superficial dermis with linear IgA with or without IgG at basement membrane

## Data Availability

All data generated or analysed during this study are included in this published article.

## LIST OF ABBREVIATIONS

IgG: Immunoglobulin G
SLE: Systemic Lupus Erythematosus

## STANDARDS OF REPORTING

CARE guidelines were followed.
